# A rapid phenotype change in the pathogen *Perkinsus marinus* was associated with a historically significant marine disease emergence in the eastern oyster

**DOI:** 10.1038/s41598-021-92379-6

**Published:** 2021-06-18

**Authors:** Ryan B. Carnegie, Susan E. Ford, Rita K. Crockett, Peter R. Kingsley-Smith, Lydia M. Bienlien, Lúcia S. L. Safi, Laura A. Whitefleet-Smith, Eugene M. Burreson

**Affiliations:** 1 Virginia Institute of Marine Science, William & Mary, P.O. Box 1346, Gloucester Point, VA 23062 USA; 2 Haskin Shellfish Research Laboratory, Rutgers University, 6959 Miller Avenue, Port Norris, NJ 08349 USA; 3 Marine Resources Research Institute, South Carolina Department of Natural Resources, 217 Fort Johnson Road, Charleston, SC 29422 USA

**Keywords:** Evolutionary ecology, Marine biology

## Abstract

The protozoan parasite *Perkinsus marinus*, which causes dermo disease in *Crassostrea virginica*, is one of the most ecologically important and economically destructive marine pathogens. The rapid and persistent intensification of dermo in the USA in the 1980s has long been enigmatic. Attributed originally to the effects of multi-year drought, climatic factors fail to fully explain the geographic extent of dermo’s intensification or the persistence of its intensified activity. Here we show that emergence of a unique, hypervirulent *P. marinus* phenotype was associated with the increase in prevalence and intensity of this disease and associated mortality. Retrospective histopathology of 8355 archival oysters from 1960 to 2018 spanning Chesapeake Bay, South Carolina, and New Jersey revealed that a new parasite phenotype emerged between 1983 and 1990, concurrent with major historical dermo disease outbreaks. Phenotypic changes included a shortening of the parasite’s life cycle and a tropism shift from deeper connective tissues to digestive epithelia. The changes are likely adaptive with regard to the reduced oyster abundance and longevity faced by *P. marinus* after rapid establishment of exotic pathogen *Haplosporidium nelsoni* in 1959. Our findings, we hypothesize, illustrate a novel ecosystem response to a marine parasite invasion: an increase in virulence in a native parasite.

## Introduction

Infectious diseases are increasing in significance for many aquatic species as human pressures on estuarine, marine, and freshwater environments intensify^1,2^, yet the factors underlying emergence of these diseases are seldom fully understood. Numerous emerging diseases have been produced by introduced exotic pathogens, such as “multinucleate sphere X” (MSX) disease caused by *Haplosporidium nelsoni* in eastern oysters, *Crassostrea virginica*, from the United States of America (USA), and bonamiasis caused by *Bonamia ostreae* in flat oysters, *Ostrea edulis*, from Europe^3,4^; infectious hematopoietic necrosis in rainbow trout, *Oncorhynchus mykiss*, in Europe^5^; and infectious hypodermal and haemopoietic necrosis, Taura syndrome, and white spot syndrome in penaeid shrimp world-wide^6^. One emerging disease has been linked to increasing local human impacts, serratiosis being transmitted to elkhorn coral *Acropora palmata* populations via wastewater from coastal communities^7^. The origins of many others are unknown, including the herpesviruses that have emerged in recent years to devastate populations of mollusc hosts like Pacific oyster *Crassostrea gigas*^8–11^.

Our objective was to better understand the re-emergence of dermo disease in the eastern oyster *Crassostrea virginica*. Dermo represents the unusual case of an established disease that was long present in a host population but which intensified sharply in the mid-1980s. This development was first apparent in the Chesapeake Bay region of the Mid-Atlantic coast of the USA. Caused by the protozoan parasite *Perkinsus marinus*, dermo historically was a chronic disease of oysters from southern estuaries of the USA into Mexico^12^. Occurring from the Gulf of Mexico north as far as Chesapeake Bay, its impacts were generally modest, with annual oyster mortality of up to 30% primarily affecting older oysters that had been exposed to the parasite for several years^13^. Beginning around 1986, dermo became an acute and profoundly destructive disease capable of causing over 70% host mortality within a period of months^14^. *P. marinus* also began a progressive range expansion northward to the northern Atlantic coast of the USA at that time, producing major disease outbreaks along the way: in Delaware Bay in 1990, where it had previously been scarcely detectable and primarily in association with oysters transplanted from the Chesapeake Bay; and in Long Island Sound and southern New England, USA, between 1991 and 1994^15^. Marine environmental change is recognized as a major driver of marine disease emergence^16,17^, and the scientific community has generally accepted the proposition that warming coastal water temperatures in recent decades, particularly in winter, promoted the range expansion of *P. marinus* poleward from the Chesapeake Bay region^15,18^.

While warming seawater temperatures may explain the range expansion of *P. marinus* and dermo disease to the north since 1990, they fail to fully explain the sudden and persistent intensification of *P. marinus* activity in Chesapeake Bay in the mid-1980s^14^. Nor do they explain an intensification of dermo disease around the same time as far south as South Carolina in the South Atlantic Bight^19^, which, in contrast with northern waters such as Delaware Bay and Long Island Sound, had not warmed significantly^20^. Elevated salinities associated with multi-year drought in the Chesapeake Bay region in the 1980s were certainly more favorable to *P. marinus* proliferation and transmission^14^. If drought was the primary cause of the intensification of dermo disease, however, the questions arise as to why more protracted and intense droughts in earlier years (see Supplementary Table S1) did not produce a similar intensification of disease; and why subsequent wet periods with depressed salinities distinctly unfavorable to *P. marinus*, for example from 2003 to 2004 (Supplementary Fig. S1), did not return the parasite to low levels of infection characteristic of earlier years. In short, environmental factors do not completely explain a lasting increase in *P. marinus* infections and associated mortality along the USA Atlantic Coast from the mid-1980s.

Dermo disease is typically detected in oysters using Ray’s fluid thioglycollate method (RFTM)^21^, a fluid culture-based technique that provides sensitive insight into the presence and abundance of *P. marinus* within infected hosts. The RFTM method does not, however, generate insights into the specific nature of the host-parasite interaction in situ or even into the size of the cells infecting the oyster, given that the parasite increases in size in its transformation from trophozoites into hypnospores in the culture medium. Paraffin histology can provide more detailed insight into host–pathogen interactions^22^, and is routinely performed for the detection of co-occurring pathogen *H. nelsoni*. *P. marinus* is generally ignored in histological diagnoses, however, in favor of the more sensitive and semi-quantitative RFTM^23^. To determine whether histology might nonetheless provide a window into the intensification of dermo disease in the 1980s, we retrieved histological slides of *P. marinus*-infected oysters which were collected from Chesapeake Bay, USA, from 1960 that were archived in a Virginia Institute of Marine Science histological collection. Then, we evaluated these slides against contemporary histology. Unexpectedly, we found a marked difference in the phenotype of *P. marinus* between slides from 1960 and the present, which prompted a wider retrospective analysis of histological samples from Chesapeake Bay spanning the entire 1960–2018 period. Histological material from New Jersey and South Carolina was added to the analysis to provide broader geographical perspective. We also drew on RFTM-based *P. marinus* infection data for Chesapeake Bay from 1952 to 2018 for additional insight into long-term infection trends. Our analyses show that while intensification of *P. marinus* infection in the mid-1980s may have been partly due to climatic factors, it was more fundamentally associated with the emergence and spread of a hypervirulent parasite phenotype.

## Results and discussion

**Figure 1:**
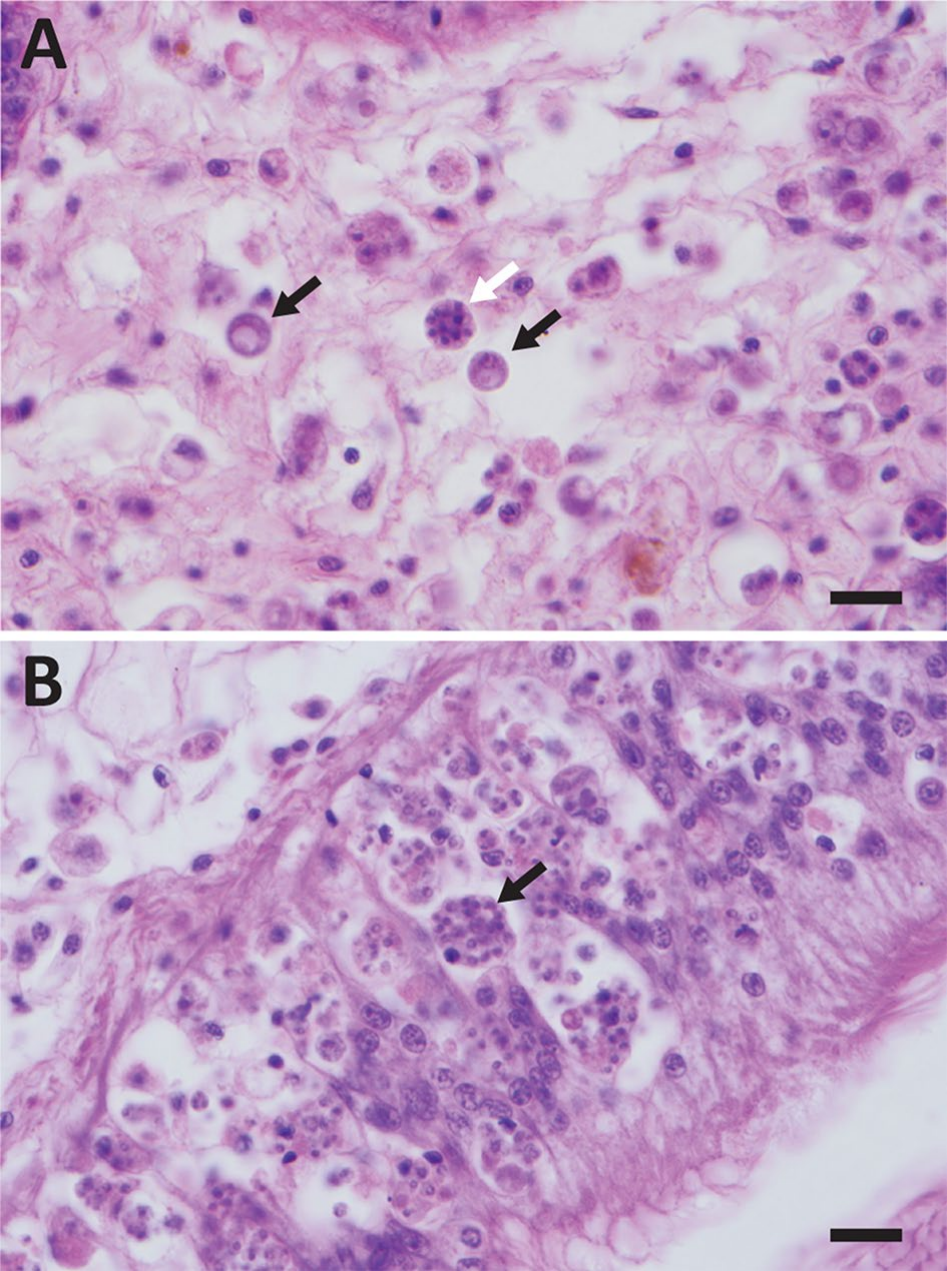
Histological presentation of *Perkinsus marinus* phenotypes. A. Original phenotype, with relatively small numbers of large *P. marinus* cells primarily infecting oyster connective tissues. Black arrows indicate two *P. marinus* trophozoites; white arrow indicates a multinucleate schizont. B. Contemporary phenotype, with large numbers of small parasite cells primarily infecting digestive epithelia. Arrow indicates a mass of *P. marinus* cells inside a moribund oyster hemocyte. Scale bars for both panels represent 20 microns.

We made two fundamental observations of changes in *P. marinus* and its interaction with eastern oysters. First, while *P. marinus* infections in early years were primarily of hemal spaces in the vesicular connective tissues that surround oyster digestive and reproductive organs, extending into mantle and gills (Fig. 1A), contemporary *P. marinus* infections are typically epithelial in tropism until they become heavy and systemic, with foci of parasite cells initially and in lighter infections observed in digestive epithelia in particular (Fig. 1B). Even in heavier pre-1980s infections, digestive epithelia were largely or entirely uncolonized by the parasite; today, *P. marinus* is primarily observed in digestive epithelia in most histological examinations.

**Figure 2:**
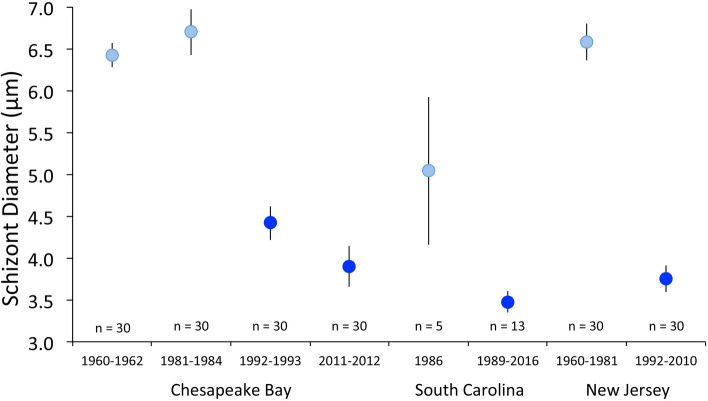
*Perkinsus marinus* schizont diameters (microns) before (light blue) and after (darker blue) the emergence of the contemporary *P. marinus* phenotype, in Chesapeake Bay, South Carolina and New Jersey. Note that for South Carolina the 1986 data represent trophozoites, as schizonts could not be definitively identified in the tissue sections. Filled circles represent means with 95% confidence intervals. Reductions in cell diameters with emergence of the contemporary phenotype within each area were statistically significant, as noted in the text. For Chesapeake Bay, Tukey multiple comparisons revealed significant cell size reductions between 1981–1984 and 1992–1993 (*P* = 0.0000000) and between 1992–1993 and 2011–2012 (*P* = 0.0050495), but not between 1960–1962 and 1981–1984 (*P* = 0.2709134).

Second, in addition to a tissue tropism distinct from that of contemporary *P. marinus,* parasite cells observed in early-era infections were much larger. Mackin et al.^24^ described cells of *P. marinus*, then identified as *Dermocystidium marinum*, to be mostly 5–7 μm in diameter, consistent with our observations of *P. marinus* in archival histology from prior to the mid-1980s (Fig. 2). *P. marinus* schizonts were found to be 6.42 ± 0.14 μm (mean ± 95% CI) in diameter in Chesapeake Bay samples from 1960 to 1962, and 6.70 ± 0.28 μm in diameter in samples from 1981–1984. *P. marinus* cells observed in samples since the mid-1980s, however, are markedly smaller: 4.42 ± 0.20 μm in diameter in 1992–1993, and 3.90 ± 0.25 μm in 2011–2012 (ANOVA *F*_0.05, 3, 116_ = 168.7, *P* < 2e−16). Samples collected from Delaware Bay showed a similar reduction in cell size, from 6.58 ± 0.22 μm in diameter in 1960–1981 to 3.75 ± 0.16 μm in 1992–2010 (Welch’s approximate *t*-test *t* = 21.53, df = 52.42, *P* < 2.2e-16), as did samples from South Carolina, which decreased in diameter from 5.04 ± 0.71 μm in diameter in 1986 to 3.48 ± 0.22 μm from 1989 to 2016 (Wilcoxon rank sum test W = 65, *P* = 0.0002334; Fig. 2). Associated with a reduction in cell size was the loss of distinctly multinucleate schizonts. While vegetative division in *P. marinus* has long been known to proceed via schizogony leading to the production of 16 or more daughter cells from a single trophozoite^25^, nuclear counts in contemporary *P. marinus* seldom exceed 2–4, indicating that the parasite may now be dividing more in a binary mode, and raising the question of whether it is still dividing by schizogony at all rather than simple binary fission.

**Figure 3:**
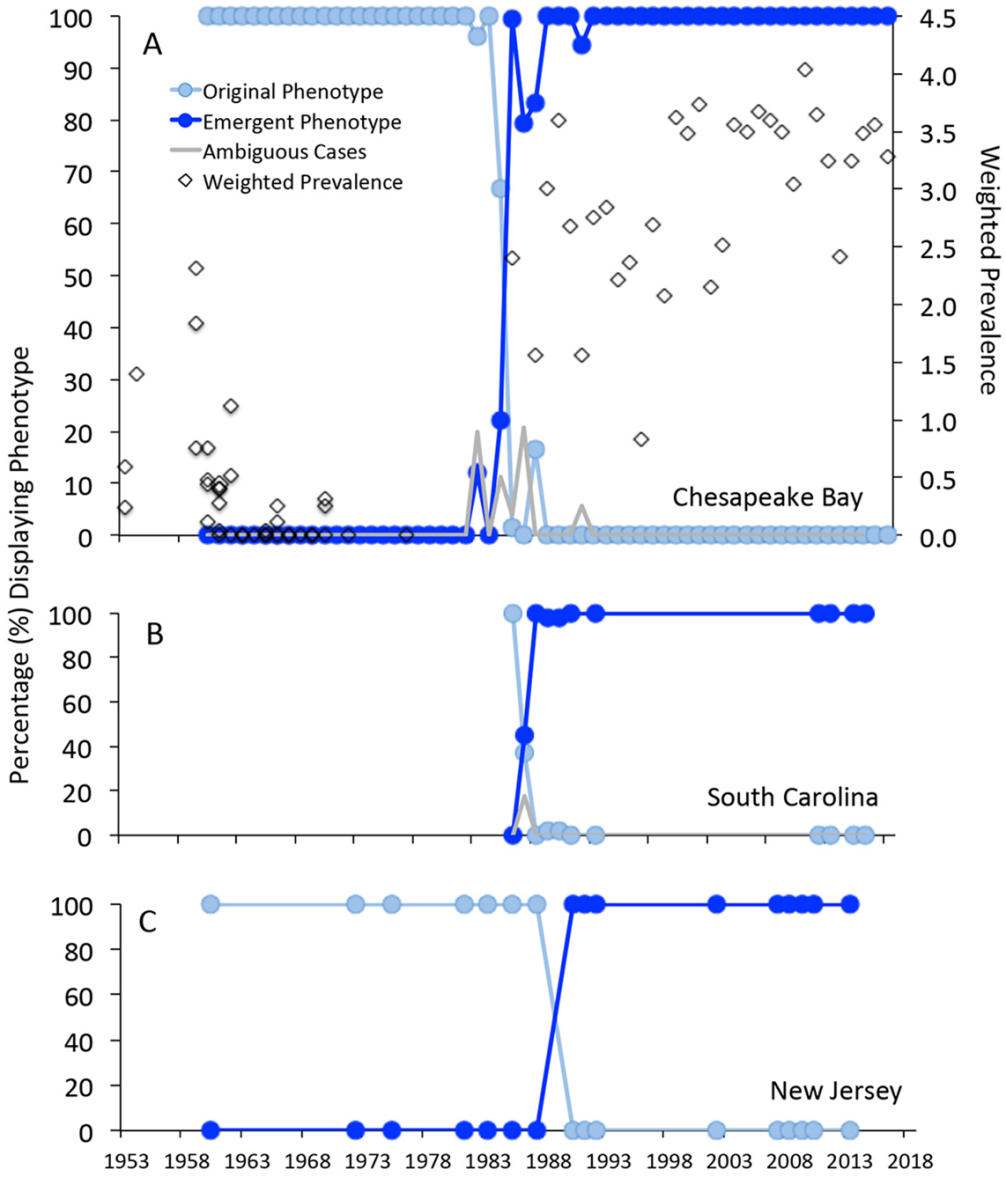
Timing of the *Perkinsus marinus* phenotype transition in Chesapeake Bay (n = 7932) (**A**), South Carolina (n = 323) (**B**) and New Jersey (n = 100) (**C**). Data presented are the percentages of analyzed oysters from each year and location displaying the original (light blue symbols and lines) or emergent (darker blue) phenotypes. The gray lines in the Chesapeake Bay and South Carolina panels represent ambiguous cases during the period of transition. For Chesapeake Bay, weighted prevalence of *P. marinus* in disease sentinel samples is also presented (diamonds), illustrating the increase in disease (as expressed in weighted prevalence) concurrent with the emergence of the contemporary phenotype. We refer the reader to Supplementary Tables S2–S4 for the annual data and sample sizes.

Using the histological observations above to define alternative parasite phenotypes—an original phenotype characterized by larger cells dividing through multinucleate schizonts and primarily infecting oyster connective tissues, and a contemporary phenotype characterized by smaller cells likely dividing in binary mode or via more oligonucleate schizonts, and primarily infecting oyster digestive epithelia—we assessed changes in the frequency of these phenotypes in oysters sampled from Chesapeake Bay as well as Delaware Bay and South Carolina between 1960 and 2018. In Chesapeake Bay, only the original phenotype was observed from 1960 to 1982 (Fig. 3A), and the contemporary phenotype has been observed exclusively since 1988. The years 1983–1988 were a transitional period, with some infections exhibiting an ambiguous phenotype, and potentially representing mixed infections. There was a major transition from the original to the contemporary phenotype, however, occurring between 1985 and 1986, between which the contemporary phenotype increased in frequency from 22.2 to 99.3% of observations. The replacement of the original phenotype with the contemporary form coincided closely with the intensification of the weighted prevalence of dermo disease (a pathogen abundance metric and traditional measure of dermo disease in a population, the sum of infection intensity scores divided by the size, n, of a sample) as measured using RFTM analyses—infection producing greater intensities of infection more rapidly than in earlier years (Fig. 3A), and associated with sharply higher mortality^14^. In South Carolina, the transition between the original and contemporary phenotypes occurred between 1986 and 1988 (Fig. 3B), coincident with increased dermo disease in oyster populations there. In Delaware Bay (Fig. 3C), no histological material was available for examination from 1988 or 1989, and only the historical phenotype was observed in limited material available for examination before that, as *P. marinus* was rarely detected in Delaware Bay before 1990. In re-analysis of samples collected from the major dermo disease outbreak in Delaware Bay in 1990, however, the contemporary phenotype was present in 100% of observations, and the original phenotype was never observed in Delaware Bay samples from subsequent years.

The picture that emerges, therefore, is of the rise of a virulent new phenotype of *P. marinus* along the Mid-Atlantic coast of the USA, which dispersed from there and supplanted a form that previously had been widely distributed in Atlantic estuaries. While changes in pathogen virulence, including virulence increases, have been documented in other systems^26^, the scope of changes to pathogen life history we have seen, including a change in tropism within the host, cell size, and potentially mode of division, occurring rapidly over a wide geographical area, is unusual. Three points should be made with regard to these phenotypic changes. First, however different the original and contemporary phenotypes appear in histological sections of oyster hosts, the evidence strongly indicates that these are the same parasite species. In situ hybridization conducted using a DNA probe designed for *P. marinus*-specificity based on DNA sequences from the contemporary phenotype^27^ unambiguously hybridized to an oyster displaying the original phenotype (Supplementary Fig. S2). While we might expect, and indeed we would hypothesize, that genetic differences underlie the respective phenotypes, these do not represent species-level differences. We also note that while genetic analyses over years have uncovered geographic differentiation of *P. marinus* populations within and between the Atlantic and Gulf^28,29^, no study has uncovered cryptic species-level diversity of *Perkinsus* parasites in oyster populations, including in the Gulf of Mexico where the original phenotype can still be observed (Supplementary Fig. S2).

The second point we would make with regard to the phenotypic changes is that they may represent a shortening of the parasite’s life cycle that would be strongly adaptive with regard to important changes in Chesapeake Bay, specifically the decrease in both oyster abundance and longevity, in the years following the emergence of *H. nelsoni* in 1959. The exotic oyster pathogen *H. nelsoni* caused mortality exceeding 90% in oysters on private Virginia aquaculture leases and public reefs in mesohaline waters by 1961^30^, resulting in a decline in oyster aquaculture production from 3,347,170 bushels in 1959 (a bushel representing approximately 200–250 oysters) to 361,792 bushels by 1983^31^. This production decline of 89% represented a depopulation of the overall aquacultured oyster population in Virginia by an estimated 1.8 billion oysters, a decrease in oyster abundance that was compounded by simultaneous reductions from wild populations that are impossible to directly quantify. A large reduction in host abundance would be detrimental to a directly transmissible pathogen entirely dependent on a single host, such as *P. marinus* in oysters. In fact, *P. marinus* did decline sharply in abundance beginning in 1959 as a likely reflection of this^32^. Recent theoretical modeling has underscored the possibility that an increase in parasite virulence could be a consequence of such reduction in host resources^33^.

Similarly detrimental to *P. marinus* as it existed from the 1950s to early 1980s would have been a reduction in oyster longevity. Wild oysters cultivated on aquaculture leases or growing on natural reefs would typically take three or more years to reach “market” size (3 inches or 76.2 mm), and many would live substantially longer, as evidenced by the larger size of oysters in historical shell middens and other deposits^34^. *P. marinus* is primarily transmitted through release into the water column with the deaths of infected hosts^35^, which in early years did not typically occur until oysters were in their third year of *P. marinus* exposure^13^. Today, high disease-caused mortality limits the age of most oysters in parasite-endemic waters to under three years^36^. *P. marinus* today contributes substantially to this contemporary mortality. The initial cause of the decrease in oyster longevity was *H. nelsoni*, however, which caused very high mortality upon its emergence^37^ and which is capable of killing oysters of a year or less in age^38^. As with the reduction in host abundance, the reduction in host longevity must have disadvantaged a directly transmissible pathogen that required several years for transmission to occur. Evolution of a parasite phenotype characterized by more rapid infection and within-host proliferation, and capable of rapidly causing high mortality, would clearly have been beneficial for exploiting the relatively sparse populations of short-lived oyster hosts that existed after the emergence of *H. nelsoni*.

The significance of the shift in parasite tropism from deeper connective tissues to more superficial digestive epithelial tissues is less clear, but it may reflect adaptation on the part of *P. marinus* to allow for gradual transmission via the feces in advance of host death^35^. This would contrast with the single release to the environment with host death that would be possible for pathogens infecting the deeper tissues, and it could be particularly advantageous when hosts are sparse. Understanding whether or not there are fitness advantages for *P. marinus* to infect digestive epithelial tissues rather than connective tissues will require further study.

Finally, the phenotypic changes in *P. marinus* highlight not only the dynamic nature of parasite evolution but also an unexplored realm of potential response to marine parasite invasion. Effects of parasite invasions are appreciated primarily with regard to impacts on “native host species, communities, and even ecosystems”^39^. Effects on native parasite communities have received little attention, yet our results suggest they may be profound. While eastern North American estuaries such as the Chesapeake Bay have seen other, eutrophication-related changes, such as increased nutrient loading and exacerbation of seasonal hypoxia with its concomitant effects on sediment biogeochemistry^40^, we cannot infer an adaptive response of *P. marinus* to these changes as we have no clear basis for relating any of these gradual environmental shifts to the parasite’s theoretical fitness. We can hypothesize, however, that the changes in *P. marinus* life history represent evolutionary adaptation to the most sudden and plausibly consequential environmental change in these estuaries, the altered abundance and demographics (longevity) of the oyster host that were produced by the invasion of *H. nelsoni*. We can only speculate as to why the emergence of the phenotype change in *P. marinus* took so long, a quarter-century after the emergence of *H. nelsoni* in Chesapeake Bay, but the delay may simply be due to the low rate of proliferation and thus mutation of *P. marinus* when hosts were sparse in the 1960s, and when hosts were still sparse and environmental conditions (particularly salinities) were unfavorable to the parasite in the 1970s (Supplementary Fig. S1). The phenotype change in the 1980s occurred when environmental conditions in Chesapeake Bay and throughout the region had become much more favorable for *P. marinus* proliferation and transmission^14^.

In summary, retrospective histological analyses have provided new insights into one of the most historically significant emergences of marine disease, the lasting increase in dermo disease in eastern oysters, identifying a rapid change in parasite virulence as associated with the disease intensification. This work underscores the importance of long-term environmental monitoring and the maintenance of associated natural history collections, without which this perspective would not have been possible.

## Methods

Eastern oysters, *C. virginica*, were collected during 1960–2018, primarily from natural oyster reefs and planting grounds, in Chesapeake Bay, South Carolina, and New Jersey. Numbers of samples collected and available for analyses varied widely. Chesapeake Bay and New Jersey samples were collected from subtidal beds by dredge, as described previously^38,41^. South Carolina samples included oysters of both subtidal and intertidal origin^42^. South Carolina collections were from locations spanning that state’s coast. New Jersey collections were from Delaware Bay, with the exception of those from 1983, 1985, and 1987, which were from the Mullica River/Great Bay system. Once collected, oysters were shucked and transverse sections of soft tissues, including stomach and intestine, digestive gland, gonad, mantle and gills, were fixed and processed for paraffin histology using standard methods, including staining with hematoxylin and eosin.

Microscopic analyses of contemporary and archival samples were performed on an Olympus BX51 light microscope using an Olympus DP71 camera. *P. marinus* phenotypes were defined after preliminary assessment of infected oyster samples from Chesapeake Bay spanning the period of the study. Phenotype A (the original phenotype) was characterized by generally larger *P. marinus* cells (> 5 μm), an abundance of multinucleate schizonts (dividing forms), and infection primarily of oyster connective tissues. Phenotype B (the contemporary phenotype) was characterized by generally smaller *P. marinus* cells (< 5 μm), a scarcity of large schizonts of nuclear count > 3 or 4, and infection primarily of oyster digestive epithelia, except in advanced, systemic cases. Across one or more samples for each area (i.e., Chesapeake Bay, South Carolina, and New Jersey) and year, data were expressed as percentage of oysters presenting either Phenotype A or Phenotype B, with some samples and years characterized by the presence of ambiguous phenotypes possibly representing mixed infection (Supplementary Tables S2–S4).

In situ hybridization was used to confirm the identity of Phenotype A as *P. marinus*. We used hybridization of *P. marinus*-specific oligonucleotide probe PmarLSU-181DIG^27^ to an oyster collected from the Gulf of Mexico coast of Florida in 2013 to determine whether a *Perkinsus* sp. displaying the original phenotype was *P. marinus*. Hybridization of *Bonamia exitiosa*-specific probe CaBon461DIG^43^ to a parallel section was used as a negative control for non-specific probe binding. Hybridization of the *P. marinus*-specific probe to a clam, *Mya arenaria*, infected with *Perkinsus chesapeaki* was included as an additional control to confirm the *P. marinus*-specificity of the reaction. Assays were conducted using an approach based on previously published protocols, with dewaxing, rehydration, permeabilization, and hybridization conducted following Carnegie et al.^44^, with the exception that pronase (125 µg/mL in phosphate buffered saline at 37 °C for 25 min) rather than proteinase K was used for permeabilization; and post-hybridization and color reaction steps conducted following Stokes and Burreson^45^. All probe concentrations were 7 ng/µL, and the *P. marinus*-specific assay included two additional unlabeled oligonucleotides at this concentration, PmarLSU-420 and PmarLSU-560 from Reece et al.^46^, to promote unfolding of the target rRNA.

For temporal perspective on the intensity of disease caused by *P. marinus* phenotypes in Chesapeake Bay, weighted prevalences of *P. marinus* infection in sentinel oyster deployments were obtained from the data archive of the long-term Virginia Institute of Marine Science Oyster Disease Monitoring Program^32,47^. Oysters from one or more low-salinity populations in Chesapeake Bay characterized by minimal or no *P. marinus* presence were deployed in spring of most years to the mesohaline York River, Virginia, where *P. marinus* infection pressure is intense. Data presented are the maximum *P. marinus* weighted prevalences in such sentinel deployments in the autumn following deployment, with weighted prevalence calculated as follows:

$$ {\text{Weighted}}\;{\text{prevalence}} = \left( {0.{\text{5}}*{\text{R}} + {\text{1}}.0*{\text{L}} + {\text{3}}.0*{\text{M}} + {\text{5}}.0*{\text{H}}} \right)/{\text{n}} $$

where R = the number of rare-intensity infections, L = the number of light-intensity infections, M = the number of moderate-intensity infections, H = the number of heavy-intensity infections, and n = the sample size analyzed, including uninfected oysters^48^ (Supplementary Table S5).

To provide a more quantitative illustration of the shift in *P. marinus* phenotypes, *P. marinus* schizont diameters were measured during selected time spans preceding and following the major transition that was apparent during the initial phase of analysis. We pooled data across years in this analysis to increase sample sizes. Temporal grouping was as narrow as possible, 2–4 years for Chesapeake Bay, but was necessarily wider for South Carolina and New Jersey because of more limited data. With the exception of South Carolina samples from 1986, schizonts were measured, rather than the generally more abundant uninucleate trophozoites which give rise to the schizonts, because schizont sizes are similar to those of mature trophozoites and do not increase with the nuclear divisions that produce the multinucleate forms^49^. As such, schizont sizes represent both the typical size of dividing stages irrespective of nuclear count and that of mature trophozoites in a given sample from a given time. The measurement of these life stages allows for straightforward comparisons among time periods. Measuring uninucleate trophozoites, on the other hand, would necessarily involve the measurement of not only mature trophozoites but also of many immature trophozoites of sub-maximal size, decreasing estimated mean diameters by degrees that could differ across samples and complicating comparisons among samples even within a time period. Trophozoites were measured for South Carolina samples from 1986 because the nature of the fixation and staining of those samples made it impossible to distinguish *P. marinus* schizonts from *H. nelsoni* plasmodia that were present in abundance. Only South Carolina trophozoites displaying vacuoplasts in their vacuoles were measured, as this structure can be considered a reasonable indicator of a mature trophozoite^49^. The smaller size and greater size variability of the cells measured from South Carolina in 1986 (see Fig. 2) illustrated the disadvantages of measuring this particular cell form. For Chesapeake Bay, pre-transition samples were analyzed in the 1960–1962 and 1981–1984 time periods; post-transition samples were analyzed from 1992–1993 and 2011–2012. For New Jersey, pre-transition samples were analyzed from 1960 to 1981, and post-transition samples spanned 1992–2010. For South Carolina, pre-transition samples were available only from 1986, while post-transition samples were evaluated from 1989 to 2016. For each location and time period, the objective was to determine mean *P. marinus* schizont diameters for thirty infected oysters, with measurement of fifteen schizonts/oyster used to calculate the means. Data were expressed as location/time period means of the individual oyster means ± SD to allow for the calculation of 95% confidence intervals for the means (Supplementary Table S6). Statistical tests to compare *P. marinus* diameters between time periods were conducted separately for each site in RStudio version 1.2.5033, R version 3.6.3 with packages tidyverse, forcats and car^50–54^: analysis of variance with Tukey’s test for multiple comparisons for Chesapeake Bay data, a Welch’s approximate *t*-test for New Jersey data, and a Wilcoxon rank sum test (because of highly uneven sample sizes) for South Carolina, with α = 0.05 for each test^55^.

To relate the temporal observations of *P. marinus* phenotypes and disease levels in Chesapeake Bay to a broader environmental trend, streamflow data were obtained from the US Geological Survey gauge on the lower James River, Virginia (37°33′47″ N, 77°32′50″ W), a major tributary of the lower Chesapeake Bay. Data were expressed as percent streamflow anomalies for mean monthly streamflow data relative to the long-term 1935–2018 trend of monthly mean flows (Supplementary Fig. S1). Positive anomalies represented months of high flow, negative anomalies months of low flow. *P. marinus* is more pathogenic at salinities above 10^56,57^, which would be more widespread during extended periods of low streamflows—i.e., periods of drought—which would cause estuarine salinities to increase. Burreson and Andrews^14^ cited multi-year drought as a key factor in the intensification of *P. marinus* activity in the 1980s (Supplementary Table S1). Periods of elevated streamflows, on the other hand, tend to depress salinities and suppress *P. marinus* activity.

## Supplementary Information

Supplementary Figure 1.

Supplementary Figure 2.

Supplementary Figure legends.

Supplementary Tables.

## Data Availability

Data are available as Supplementary Information. Histological materials are available on request via VIMS, the Haskin Shellfish Research Laboratory (Rutgers), and the South Carolina Department of Natural Resources.
