# Supplementary figures and images for: A rapid phenotype change in the pathogen Perkinsus marinus was associated with a historically significant marine disease emergence in the eastern oyster
Source: Sci Rep. 2021 Jun 18;11:12872. doi: 10.1038/s41598-021-92379-6 (PMC8213716; doi:10.1038/s41598-021-92379-6)

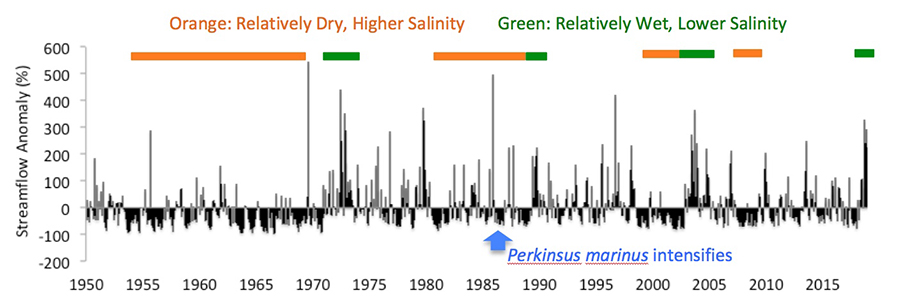

Supplement: Supplementary file 1 — Supplementary Figure 1. [file 41598_2021_92379_MOESM1_ESM.jpg]

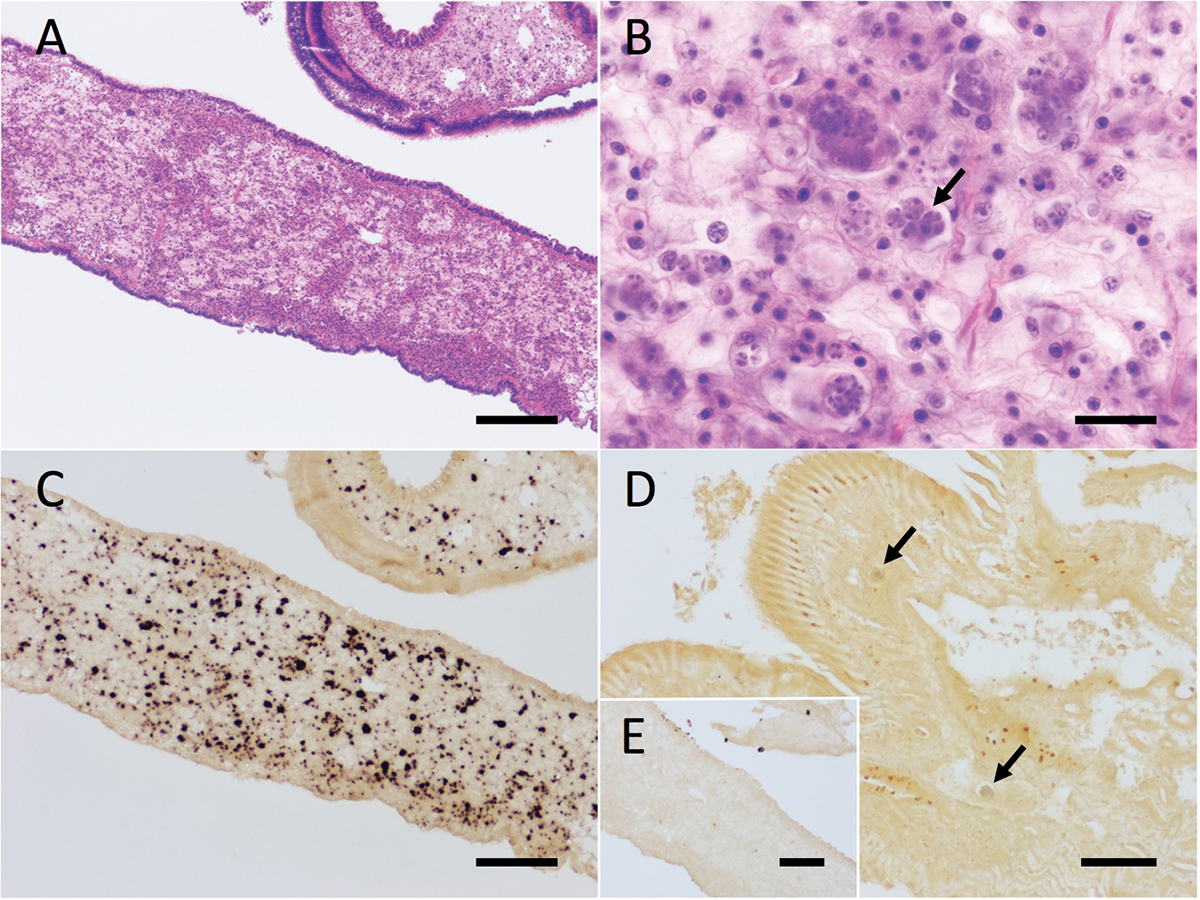

Supplement: Supplementary file 2 — Supplementary Figure 2. [file 41598_2021_92379_MOESM2_ESM.jpg]
