# Supplementary material for: A rapid phenotype change in the pathogen Perkinsus marinus was associated with a historically significant marine disease emergence in the eastern oyster
Source: Sci Rep. 2021 Jun 18;11:12872. doi: 10.1038/s41598-021-92379-6 (PMC8213716; doi:10.1038/s41598-021-92379-6)
Supplement: Supplementary file 3 — Supplementary Figure legends. [file 41598_2021_92379_MOESM3_ESM.docx]

**Supplementary Figure Legends**

**Supplementary Fig. 1.** Monthly streamflow anomaly trend for the James River, Virginia, 1950-2018. The mid-1980s intensification of *Perkinsus marinus* activity has been attributed to increased salinities during multi-year drought in 1980-1982 and 1985-1987 [14], indicated by extended periods of negative streamflow anomalies. Reduced salinities more unfavorable to *P. marinus* are associated with periods of high streamflows, indicated by positive anomalies.

**Supplementary Fig. 2.** *In situ* hybridization for *Perkinsus marinus* displaying original phenotype. A. Hematoxylin-and-eosin-stained section of oyster mantle showing the infiltration of the tissue by *P. marinus* cells and oyster hemocytes. Scale bar = 100 microns. B. Higher magnification of *P. marinus* infection of oyster mantle, arrow highlighting a cluster of three *P. marinus* schizonts. Scale bar = 20 microns. C. Successful hybridization of *P. marinus*-specific DNA probes to the same section as A, dark areas representing probe binding to *P. marinus* cells. Scale bar = 100 microns. D. Lack of hybridization of *P. marinus*-specific DNA probes to a section of *Mya arenaria* infected with congeneric parasite *Perkinsus chesapeaki*, demonstrating the specificity of the *P. marinus* assay. Arrows indicate clusters of *P. chesapeaki* cells not reacting with the *P. marinus*-specific probes. Scale bar = 100 microns. E. Lack of hybridization of negative control *Bonamia exitiosa*-specific probes to the *P. marinus* section, providing further evidence for the specificity of the *P. marinus* assay, probes not complementary to (specific for) *P. marinus* failing to hybridize to *P. marinus* in the section.
