# Supplementary material for: A rapid phenotype change in the pathogen Perkinsus marinus was associated with a historically significant marine disease emergence in the eastern oyster
Source: Sci Rep. 2021 Jun 18;11:12872. doi: 10.1038/s41598-021-92379-6 (PMC8213716; doi:10.1038/s41598-021-92379-6)
Supplement: Supplementary file 4 — Supplementary Tables. [file 41598_2021_92379_MOESM4_ESM.docx]

**SUPPLEMENTARY INFORMATION**

Carnegie, Ford, Crockett, Kingsley-Smith, Bienlien, Safi, Whitefleet-Smith, and Burreson,

“A Rapid Phenotype Change in the Pathogen *Perkinsus marinus* Was Associated with a Historically Significant Marine Disease Emergence in the Eastern Oyster”

Six supplementary data tables

Two supplementary figures

**Supplementary Table 1.** Significant drought periods preceding the intensification of disease in eastern oysters, as reflected in streamflow anomalies recorded in the James River, Virginia, as reported in US Geological Survey streamflow records for the James River at Richmond, station 02037500. Multi-year droughts, to which Burreson and Andrews [14] attributed the 1980s intensification of disease, are indicated with asterisks.

| **Period** | **Overall Duration (Months)** | **Months of Below-Average Streamflow** | **Mean Negative Streamflow Anomaly** |
| --- | --- | --- | --- |
| **1953-57** | **56** | **49** | **-51.0** |
| **1963-66** | **42** | **38** | **-66.4** |
| **1966-69** | **32** | **29** | **-47.9** |
| **1980-82*** | **20** | **19** | **-54.4** |
| **1984-87*** | **29** | **24** | **-40.0** |

**Supplementary Table 2.** *Perkinsus marinus* phenotypes in Chesapeake Bay oysters.

| **Year** | **Overall** | **Phenotype A-Original** | | **Phenotype B-Contemporary** | | **Ambiguous Cases** | |
| --- | --- | --- | --- | --- | --- | --- | --- |
|  | **N** | **n** | **%** | **n** | **%** | **n** | **%** |
| 1960 | 19 | 19 | 100.0 | 0 | 0.0 | 0 | 0.0 |
| 1961 | 20 | 20 | 100.0 | 0 | 0.0 | 0 | 0.0 |
| 1962 | 30 | 30 | 100.0 | 0 | 0.0 | 0 | 0.0 |
| 1963 | 18 | 18 | 100.0 | 0 | 0.0 | 0 | 0.0 |
| 1964 | 30 | 30 | 100.0 | 0 | 0.0 | 0 | 0.0 |
| 1965 | 38 | 38 | 100.0 | 0 | 0.0 | 0 | 0.0 |
| 1966 | 27 | 27 | 100.0 | 0 | 0.0 | 0 | 0.0 |
| 1967 | 18 | 18 | 100.0 | 0 | 0.0 | 0 | 0.0 |
| 1968 | 29 | 29 | 100.0 | 0 | 0.0 | 0 | 0.0 |
| 1969 | 39 | 39 | 100.0 | 0 | 0.0 | 0 | 0.0 |
| 1970 | 17 | 17 | 100.0 | 0 | 0.0 | 0 | 0.0 |
| 1971 | 26 | 26 | 100.0 | 0 | 0.0 | 0 | 0.0 |
| 1972 | 30 | 30 | 100.0 | 0 | 0.0 | 0 | 0.0 |
| 1973 | 39 | 39 | 100.0 | 0 | 0.0 | 0 | 0.0 |
| 1974 | 17 | 17 | 100.0 | 0 | 0.0 | 0 | 0.0 |
| 1975 | 21 | 21 | 100.0 | 0 | 0.0 | 0 | 0.0 |
| 1976 | 22 | 22 | 100.0 | 0 | 0.0 | 0 | 0.0 |
| 1977 | 34 | 34 | 100.0 | 0 | 0.0 | 0 | 0.0 |
| 1978 | 18 | 18 | 100.0 | 0 | 0.0 | 0 | 0.0 |
| 1979 | 39 | 39 | 100.0 | 0 | 0.0 | 0 | 0.0 |
| 1980 | 26 | 26 | 100.0 | 0 | 0.0 | 0 | 0.0 |
| 1981 | 16 | 16 | 100.0 | 0 | 0.0 | 0 | 0.0 |
| 1982 | 2 | 2 | 100.0 | 0 | 0.0 | 0 | 0.0 |
| 1983 | 25 | 24 | 96.0 | 3 | 12.0 | 5 | 20.0 |
| 1984 | 4 | 4 | 100.0 | 0 | 0.0 | 0 | 0.0 |
| 1985 | 18 | 12 | 66.7 | 4 | 22.2 | 2 | 11.1 |
| 1986 | 149 | 2 | 1.3 | 148 | 99.3 | 6 | 4.0 |
| 1987 | 29 | 0 | 0.0 | 23 | 79.3 | 6 | 20.7 |
| 1988 | 24 | 4 | 16.7 | 20 | 83.3 | 0 | 0.0 |
| 1989 | 21 | 0 | 0.0 | 21 | 100.0 | 0 | 0.0 |
| 1990 | 20 | 0 | 0.0 | 20 | 100.0 | 0 | 0.0 |
| 1991 | 23 | 0 | 0.0 | 23 | 100.0 | 0 | 0.0 |
| 1992 | 18 | 0 | 0.0 | 17 | 94.4 | 1 | 5.6 |
| 1993 | 19 | 0 | 0.0 | 19 | 100.0 | 0 | 0.0 |
| 1994 | 22 | 0 | 0.0 | 22 | 100.0 | 0 | 0.0 |
| 1995 | 21 | 0 | 0.0 | 21 | 100.0 | 0 | 0.0 |
| 1996 | 22 | 0 | 0.0 | 22 | 100.0 | 0 | 0.0 |
| 1997 | 20 | 0 | 0.0 | 20 | 100.0 | 0 | 0.0 |
| 1998 | 22 | 0 | 0.0 | 22 | 100.0 | 0 | 0.0 |
| 1999 | 21 | 0 | 0.0 | 21 | 100.0 | 0 | 0.0 |
| 2000 | 20 | 0 | 0.0 | 20 | 100.0 | 0 | 0.0 |
| 2001 | 23 | 0 | 0.0 | 23 | 100.0 | 0 | 0.0 |
| 2002 | 484 | 0 | 0.0 | 484 | 100.0 | 0 | 0.0 |
| 2003 | 188 | 0 | 0.0 | 188 | 100.0 | 0 | 0.0 |
| 2004 | 185 | 0 | 0.0 | 185 | 100.0 | 0 | 0.0 |
| 2005 | 230 | 0 | 0.0 | 230 | 100.0 | 0 | 0.0 |
| 2006 | 155 | 0 | 0.0 | 155 | 100.0 | 0 | 0.0 |
| 2007 | 263 | 0 | 0.0 | 263 | 100.0 | 0 | 0.0 |
| 2008 | 527 | 0 | 0.0 | 527 | 100.0 | 0 | 0.0 |
| 2009 | 706 | 0 | 0.0 | 706 | 100.0 | 0 | 0.0 |
| 2010 | 703 | 0 | 0.0 | 703 | 100.0 | 0 | 0.0 |
| 2011 | 642 | 0 | 0.0 | 642 | 100.0 | 0 | 0.0 |
| 2012 | 472 | 0 | 0.0 | 472 | 100.0 | 0 | 0.0 |
| 2013 | 399 | 0 | 0.0 | 399 | 100.0 | 0 | 0.0 |
| 2014 | 308 | 0 | 0.0 | 308 | 100.0 | 0 | 0.0 |
| 2015 | 334 | 0 | 0.0 | 334 | 100.0 | 0 | 0.0 |
| 2016 | 316 | 0 | 0.0 | 316 | 100.0 | 0 | 0.0 |
| 2017 | 341 | 0 | 0.0 | 341 | 100.0 | 0 | 0.0 |
| 2018 | 233 | 0 | 0.0 | 233 | 100.0 | 0 | 0.0 |

**Supplementary Table 3.** *Perkinsus marinus* phenotypes in New Jersey oysters.

| **Year** | **Overall** | **Phenotype A-Original** | | **Phenotype B-Contemporary** | | **Ambiguous Cases** | |
| --- | --- | --- | --- | --- | --- | --- | --- |
|  | **N** | **n** | **%** | **n** | **%** | **n** | **%** |
| 1960 | 1 | 1 | 100.0 | 0 | 0.0 | 0 | 0.0 |
| 1961 | 0 |  |  |  |  |  |  |
| 1962 | 0 |  |  |  |  |  |  |
| 1963 | 0 |  |  |  |  |  |  |
| 1964 | 0 |  |  |  |  |  |  |
| 1965 | 0 |  |  |  |  |  |  |
| 1966 | 0 |  |  |  |  |  |  |
| 1967 | 0 |  |  |  |  |  |  |
| 1968 | 0 |  |  |  |  |  |  |
| 1969 | 0 |  |  |  |  |  |  |
| 1970 | 0 |  |  |  |  |  |  |
| 1971 | 0 |  |  |  |  |  |  |
| 1972 | 29 | 29 | 100.0 | 0 | 0.0 | 0 | 0.0 |
| 1973 | 0 |  |  |  |  |  |  |
| 1974 | 0 |  |  |  |  |  |  |
| 1975 | 10 | 10 | 100.0 | 0 | 0.0 | 0 | 0.0 |
| 1976 | 0 |  |  |  |  |  |  |
| 1977 | 0 |  |  |  |  |  |  |
| 1978 | 0 |  |  |  |  |  |  |
| 1979 | 0 |  |  |  |  |  |  |
| 1980 | 0 |  |  |  |  |  |  |
| 1981 | 1 | 1 | 100.0 | 0 | 0.0 | 0 | 0.0 |
| 1982 | 0 |  |  |  |  |  |  |
| 1983 | 1 | 1 | 100.0 | 0 | 0.0 | 0 | 0.0 |
| 1984 | 0 |  |  |  |  |  |  |
| 1985 | 2 | 2 | 100.0 | 0 | 0.0 | 0 | 0.0 |
| 1986 | 0 |  |  |  |  |  |  |
| 1987 | 1 | 1 | 100.0 | 0 | 0.0 | 0 | 0.0 |
| 1988 | 0 |  |  |  |  |  |  |
| 1989 | 0 |  |  |  |  |  |  |
| 1990 | 10 | 0 | 0.0 | 10 | 100.0 | 0 | 0.0 |
| 1991 | 1 | 0 | 0.0 | 1 | 100.0 | 0 | 0.0 |
| 1992 | 16 | 0 | 0.0 | 16 | 100.0 | 0 | 0.0 |
| 1993 | 0 |  |  |  |  |  |  |
| 1994 | 0 |  |  |  |  |  |  |
| 1995 | 0 |  |  |  |  |  |  |
| 1996 | 0 |  |  |  |  |  |  |
| 1997 | 0 |  |  |  |  |  |  |
| 1998 | 0 |  |  |  |  |  |  |
| 1999 | 0 |  |  |  |  |  |  |
| 2000 | 0 |  |  |  |  |  |  |
| 2001 | 0 |  |  |  |  |  |  |
| 2002 | 4 | 0 | 0.0 | 4 | 100.0 | 0 | 0.0 |
| 2003 | 0 |  |  |  |  |  |  |
| 2004 | 0 |  |  |  |  |  |  |
| 2005 | 0 |  |  |  |  |  |  |
| 2006 | 0 |  |  |  |  |  |  |
| 2007 | 3 | 0 | 0.0 | 3 | 100.0 | 0 | 0.0 |
| 2008 | 15 | 0 | 0.0 | 15 | 100.0 | 0 | 0.0 |
| 2009 | 2 | 0 | 0.0 | 2 | 100.0 | 0 | 0.0 |
| 2010 | 3 | 0 | 0.0 | 3 | 100.0 | 0 | 0.0 |
| 2011 | 0 |  |  |  |  |  |  |
| 2012 | 0 |  |  |  |  |  |  |
| 2013 | 1 | 0 | 0.0 | 1 | 100.0 | 0 | 0.0 |

**Supplementary Table 4.** *Perkinsus marinus* phenotypes in South Carolina oysters.

| **Year** | **Overall** | **Phenotype A-Original** | | **Phenotype B-Contemporary** | | **Ambiguous Cases** | |
| --- | --- | --- | --- | --- | --- | --- | --- |
|  | **N** | **n** | **%** | **n** | **%** | **n** | **%** |
| 1986 | 36 | 36 | 100.0 | 0 | 0.0 | 0 | 0.0 |
| 1987 | 51 | 19 | 37.3 | 23 | 45.1 | 9 | 17.6 |
| 1988 | 28 | 0 | 0.0 | 28 | 100.0 | 0 | 0.0 |
| 1989 | 47 | 1 | 2.1 | 46 | 97.9 | 0 | 0.0 |
| 1990 | 54 | 1 | 1.9 | 53 | 98.1 | 0 | 0.0 |
| 1991 | 27 | 0 | 0.0 | 27 | 100.0 | 0 | 0.0 |
| 1992 | 0 |  |  |  |  |  |  |
| 1993 | 12 | 0 | 0.0 | 12 | 100.0 | 0 | 0.0 |
| • |  |  |  |  |  |  |  |
| • |  |  |  |  |  |  |  |
| • |  |  |  |  |  |  |  |
| 2012 | 5 | 0 | 0.0 | 5 | 100.0 | 0 | 0.0 |
| 2013 | 10 | 0 | 0.0 | 10 | 100.0 | 0 | 0.0 |
| • |  |  |  |  |  |  |  |
| • |  |  |  |  |  |  |  |
| • |  |  |  |  |  |  |  |
| 2016 | 53 | 0 | 0.0 | 53 | 100.0 | 0 | 0.0 |

**Supplementary Table 5.** Maximum autumn weighted prevalences of *Perkinsus marinus* in sentinel oysters deployed in spring to the York River, Chesapeake Bay, Virginia, 1953-2018.

| **Year(s)** | **Sentinel Deployments** | **Weighted Prevalence(s)** |
| --- | --- | --- |
|  | **N** |  |
| 1953 | 2 | 0.60, 0.24 |
| 1954 | 1 | 1.40 |
| 1955-1958 | 0 |  |
| 1959 | 3 | 2.32, 1.84, 0.76 |
| 1960 | 4 | 0.48, 0.12, 0.44, 0.76 |
| 1961 | 6 | 0.00, 0.04, 0.28, 0.40, 0.44, 0.40 |
| 1962 | 3 | 0.00, 0.52, 1.12 |
| 1963 | 2 | 0.00, 0.00 |
| 1964 | 1 | 0.00 |
| 1965 | 3 | 0.00, 0.04, 0.00 |
| 1966 | 3 | 0.26, 0.00, 0.12 |
| 1967 | 2 | 0.00, 0.00 |
| 1968 | 1 | 0.00 |
| 1969 | 2 | 0.00, 0.00 |
| 1970 | 3 | 0.00, 0.25, 0.32 |
| 1971 | 0 |  |
| 1972 | 1 | 0.00 |
| 1973-1976 | 0 |  |
| 1977 | 1 | 0.00 |
| 1978-1985 | 0 |  |
| 1986 | 1 | 2.40 |
| 1987 | 0 |  |
| 1988 | 1 | 1.56 |
| 1989 | 1 | 3.00 |
| 1990 | 1 | 3.60 |
| 1991 | 1 | 2.68 |
| 1992 | 1 | 1.56 |
| 1993 | 1 | 2.76 |
| 1994 | 1 | 2.84 |
| 1995 | 1 | 2.22 |
| 1996 | 1 | 2.36 |
| 1997 | 1 | 0.83 |
| 1998 | 1 | 2.69 |
| 1999 | 1 | 2.08 |
| 2000 | 1 | 3.62 |
| 2001 | 1 | 3.48 |
| 2002 | 1 | 3.73 |
| 2003 | 1 | 2.15 |
| 2004 | 1 | 2.52 |
| 2005 | 1 | 3.56 |
| 2006 | 1 | 3.50 |
| 2007 | 1 | 3.67 |
| 2008 | 1 | 3.60 |
| 2009 | 1 | 3.50 |
| 2010 | 1 | 3.04 |
| 2011 | 1 | 4.04 |
| 2012 | 1 | 3.64 |
| 2013 | 1 | 3.24 |
| 2014 | 1 | 2.42 |
| 2015 | 1 | 3.13 |
| 2016 | 1 | 3.48 |
| 2017 | 1 | 3.56 |
| 2018 | 1 | 3.28 |

**Supplementary Table 6.** Change in cell size of *Perkinsus marinus* schizonts from Chesapeake Bay and New Jersey, with data from South Carolina on mature trophozoites (1986) and schizonts (1989-2016). Numbers in parenthesis indicate sample sizes where fewer (or more) than fifteen *P. marinus* schizonts could be measured.

|  | **Chesapeake Bay** | | | |  | **New Jersey** | |  | **South Carolina** | |
| --- | --- | --- | --- | --- | --- | --- | --- | --- | --- | --- |
|  | **1960-1962** | **1981-1984** | **1992-1993** | **2011-2012** |  | **1960-1981** | **1992-2010** |  | **1986** | **1989-2016** |
| 1 | 6.11 | 7.67 | 5.55 | 3.73 |  | 7.04 | 4.49 |  | 5.38 | 3.19 |
| 2 | 6.84 (7) | 5.72 | 4.54 (3) | 3.57 |  | 5.99 (8) | 3.50 |  | 4.69 | 3.61 (9) |
| 3 | 6.31 (14) | 6.52 | 5.06 | 3.86 |  | 6.72 | 2.95 (12) |  | 4.43 | 3.73 |
| 4 | 6.02 | 9.27 (10) | 4.46 | 3.70 |  | 5.37 | 3.07 |  | 4.58 | 3.21 (4) |
| 5 | 6.94 | 7.68 | 4.61 | 3.87 (10) |  | 7.44 (14) | 3.54 |  | 6.14 | 3.60 (19) |
| 6 | 7.08 | 6.23 | 4.67 | 3.52 |  | 7.54 | 3.74 |  |  | 3.52 |
| 7 | 6.55 (10) | 5.98 | 3.96 | 3.68 |  | 6.76 | 3.07 |  |  | 3.84 |
| 8 | 6.22 | 6.61 | 4.75 | 3.72 (5) |  | 6.41 | 3.98 |  |  | 3.40 (13) |
| 9 | 6.56 | 6.34 | 4.50 | 3.97 (5) |  | 6.29 (2) | 3.97 (12) |  |  | 3.17 (9) |
| 10 | 6.41 | 6.14 | 3.87 | 3.39 |  | 6.52 (7) | 3.98 (5) |  |  | 3.34 (14) |
| 11 | 6.64 | 6.24 | 3.62 (12) | 4.05 |  | 6.75 | 3.85 (6) |  |  | 3.35 |
| 12 | 6.66 | 6.08 | 4.70 | 3.18 |  | 6.59 | 4.16 |  |  | 3.65 |
| 13 | 6.13 | 6.18 | 3.93 (5) | 3.00 |  | 6.26 | 3.84 |  |  | 3.58 |
| 14 | 6.16 | 6.13 | 5.35 | 3.62 |  | 6.21 | 4.37 |  |  |  |
| 15 | 6.26 | 6.63 | 3.83 | 4.28 |  | 6.44 (8) | 4.07 |  |  |  |
| 16 | 7.15 | 6.74 | 3.50 | 3.98 |  | 5.59 | 3.92 |  |  |  |
| 17 | 5.94 | 6.43 | 4.81 | 3.71 |  | 6.80 | 3.73 |  |  |  |
| 18 | 6.85 (11) | 6.14 | 3.35 | 3.72 |  | 5.57 | 4.22 |  |  |  |
| 19 | 6.24 (13) | 6.77 | 3.89 | 4.74 |  | 5.91 | 3.63 |  |  |  |
| 20 | 6.24 | 6.90 (10) | 4.62 | 4.72 |  | 6.19 | 3.77 |  |  |  |
| 21 | 5.59 | 6.16 (10) | 3.76 | 4.53 |  | 7.02 | 3.57 |  |  |  |
| 22 | 6.70 | 7.78 | 4.56 | 6.34 |  | 7.27 | 2.66 |  |  |  |
| 23 | 6.09 | 6.85 (6) | 4.88 | 4.87 |  | 6.55 | 3.86 |  |  |  |
| 24 | 5.89 | 6.22 | 4.31 | 3.87 |  | 7.58 | 3.91 (13) |  |  |  |
| 25 | 6.19 | 6.11 | 4.68 | 3.78 |  | 7.66 (3) | 3.46 |  |  |  |
| 26 | 6.18 | 7.01 | 5.14 (13) | 4.09 |  | 6.34 | 3.67 |  |  |  |
| 27 | 6.73 | 6.63 | 4.66 | 3.06 |  | 6.89 | 3.60 |  |  |  |
| 28 | 6.62 | 6.92 | 4.18 | 3.39 |  | 6.31 | 3.71 |  |  |  |
| 29 | 6.88 | 7.62 (4) | 4.22 | 3.77 |  | 6.31 | 3.75 |  |  |  |
| 30 | 6.49 | 7.33 (2) | 4.59 | 3.19 |  | 7.14 | 4.47 |  |  |  |
| n | 30 | 30 | 30 | 30 |  | 30 | 30 |  | 5 | 13 |
| Mean | 6.42 | 6.70 | 4.42 | 3.90 |  | 6.58 | 3.75 |  | 5.04 | 3.48 |
| SD | 0.38 | 0.74 | 0.54 | 0.66 |  | 0.59 | 0.42 |  | 0.71 | 0.22 |
